# Preliminary evidence that different mechanisms underlie the anger superiority effect in children with and without Autism Spectrum Disorders

**DOI:** 10.3389/fpsyg.2014.00461

**Published:** 2014-05-27

**Authors:** Tomoko Isomura, Shino Ogawa, Satoko Yamada, Masahiro Shibasaki, Nobuo Masataka

**Affiliations:** Primate Research Institute, Kyoto UniversityInuyama, Japan

**Keywords:** Autism Spectrum Disorders, anger superiority effect, children, face-in-the-crowd effect, visual search, emotion, facial expressions, attention

## Abstract

Previous studies have demonstrated that angry faces capture humans' attention more rapidly than emotionally positive faces. This phenomenon is referred to as the anger superiority effect (ASE). Despite atypical emotional processing, adults and children with Autism Spectrum Disorders (ASD) have been reported to show ASE as well as typically developed (TD) individuals. So far, however, few studies have clarified whether or not the mechanisms underlying ASE are the same for both TD and ASD individuals. Here, we tested how TD and ASD children process schematic emotional faces during detection by employing a recognition task in combination with a face-in-the-crowd task. Results of the face-in-the-crowd task revealed the prevalence of ASE both in TD and ASD children. However, the results of the recognition task revealed group differences: In TD children, detection of angry faces required more configural face processing and disrupted the processing of local features. In ASD children, on the other hand, it required more feature-based processing rather than configural processing. Despite the small sample sizes, these findings provide preliminary evidence that children with ASD, in contrast to TD children, show quick detection of angry faces by extracting local features in faces.

## Introduction

The ability to detect threatening social stimuli quickly and modify our behaviors according to the context is beneficial for avoiding social conflict. Our visual system is, therefore, thought to have evolved to be more sensitive to threatening faces than to other facial expressions (Ohman and Soares, 1993; Ohman et al., 2001). Angry faces are universally treated as signals of potential threat. They are processed rapidly and efficiently, and are particularly efficient in capturing attention (Vuilleumier and Schwartz, 2001). This phenomenon is defined as the anger superiority effect (ASE). ASE has been studied using a visual search paradigm in which participants searched for discrepant angry or happy faces in a crowd of distractor faces (i.e., Face-in-the-crowd task; Hansen and Hansen, 1988; Horstmann and Bauland, 2006; Pinkham et al., 2010). Several studies have confirmed that ASE can also be observed with schematic-faces (Fox et al., 2000; Eastwood et al., 2001; Ohman et al., 2001; Horstmann, 2009). By using schematic faces it is possible to eliminate many low-level perceptual variations found in photographs of emotional expressions, and to better control experiment variables.

ASE has recently been tested in participants with Autism Spectrum Disorders (ASD) using the face-in-the-crowd paradigm in adults (Ashwin et al., 2006; Krysko and Rutherford, 2009), as well as children and adolescents (Rosset et al., 2011; Isomura et al., submitted). ASD are neurodevelopmental disorders characterized by social communicative difficulties and restricted behaviors and interests (American Psychiatric Association, 2013). Previous studies have reported that individuals with ASD show specific difficulties in social and emotional information processing (Dawson et al., 2005). In particular an atypical pattern of face processing has often been reported: while TD individuals tend to use a configural style for face processing (Tanaka and Farah, 1993), individuals with ASD have been shown to have difficulties in configural processing and to focus more on local features in faces (Behrmann et al., 2006). Also, recent studies revealed that individuals with ASD showed atypical emotional responses to faces, in which undifferentiated affective responses were observed to different facial emotions in event-related potentials (ERPs) responses (Wagner et al., 2013) as well as facial electromyography (EMG) activities (McIntosh et al., 2006; Beall et al., 2008; Rozga et al., 2013).

Contrary to their atypical cognitive processing and emotional responses to facial emotions, however, recent studies have revealed that ASE exists in most of the population with ASD as well as TD individuals (Ashwin et al., 2006; Krysko and Rutherford, 2009; Rosset et al., 2011; Isomura et al., submitted). Interestingly though, it has been consistently reported that ASE in ASD was not as robust as that in TD individuals. Individuals with ASD did not show the effect when a large number of distractor faces (crowd size) was presented. (Ashwin et al., 2006; Krysko and Rutherford, 2009; Isomura et al., submitted). In addition, Isomura et al. (submitted) found age differences in ASE only in ASD but not in TD. These findings suggest that individuals with ASD employed compensatory but less-effective mechanisms that might be learned/acquired in their development.

Previous studies using schematic face stimuli in TD individuals have suggested that ASE requires configural/holistic level of face-processing, because the effect was not seen when threatening single features were presented in isolation (Fox et al., 2000; Tipples et al., 2002; Weymar et al., 2011). There are, however, no studies on individuals with ASD that examined the cognitive mechanisms underlying ASE. Therefore, we aimed to directly examine the cognitive mechanisms underlying ASE in ASD in order to understand how individuals with ASD compensatorily develop/acquire the mechanisms to process social threat rapidly. Given the atypical pattern of face processing in individuals with ASD (Behrmann et al., 2006), they may extract facial information from local features, rather than using higher level configural processing in detecting emotional faces (Ashwin et al., 2006; Behrmann et al., 2006; Krysko and Rutherford, 2009).

In the current study, we examined whether ASD and TD participants employed a configural processing or a feature-based processing during a face-in-the-crowd task. Here we employed a recognition task in combination with the face-in-the-crowd task. A recent study revealed that humans' cognitive tendency toward configural processing of faces reduces their ability to recognize differences of local features (Wilford and Wells, 2010). In our study, therefore, we had expected that the cognitive pattern that participants employ during a face-in-the-crowd task would be reflected in their performance of the recognition task. We used whole faces, local features with outline of a face, and inverted faces for recognition. Inverted faces are well known to disrupt configural processing (Yin, 1969) while they include same volume of physical information as the whole (upright) faces. Thus, we had expected that participants would show poorer performance on recognition of both local features and inverted faces if they relied on the configural processing when searching.

Given that the previous study showed that children with ASD aged 9–10 years old started to show ASE (Isomura et al., submitted), we focused on children with an average age of about 10 years old in the current study. We hypothesized that TD children would show better performance in recognizing the whole face rather than local features or inverted faces based on previous studies (Tanaka and Farah, 1993; Wilford and Wells, 2010). In addition, TD children would show better performance in recognizing local features in *happy* faces then those in *angry* faces according to a previous study showing that negative facial expressions disrupt the processing of local features (Eastwood et al., 2008). In ASD children, on the other hand, we hypothesized that they would show similar performance in recognizing whole faces, local features, and inverted faces because individuals with ASD may focus on local features during the face-in-the-crowd task.

## Methods

### Ethics note

This study was ethically reviewed by the institutional ethics committee of experiments for human participants prior to the study (permission number, #H2012-05). We adhered to the Declaration of Helsinki and the institutional guidelines for experiments with human participants.

### Participants

Twenty children with ASD (16 male and 4 female) and 22 typically developing children (18 male and 4 female) participated in this study. The participants in the ASD group were diagnosed either with Pervasive Developmental Disorder (3 children), Autism Spectrum Disorder (9), Asperger's syndrome (5), High-functioning Autism (2), or Pervasive Developmental Disorder—Not Otherwise Specified (1) by child psychiatrists based on either DSM-IV or ICD10. Subjects have been participating in the Developmental Disorders and Support for Acquiring Reading and Writing Skills project at the Kokoro Research Center in Kyoto University. Children with no history of any psychiatric condition were recruited via the local community as a control group.

Intelligence Quotient (IQ) was measured using the Japanese version of the Wechsler Intelligence Scale for Children (either WISC-III or WISC-IV). Subjects' parents answered the Japanese version of the Autism Spectrum Quotient (AQ) (Wakabayashi et al., 2006). To be included in the ASD group, participants had to meet the criteria of AQ with a score more than 20, and to be included in the TD group, they had to meet the criteria of AQ with a score less than 20, according to the cut-off criteria established by Wakabayashi et al. (2006). Additionally, participants had to meet the criteria of IQ with a score of 70 or higher for both groups.

One individual in the ASD group and 4 individuals in the TD group were excluded from analysis because they did not meet the criteria of AQ. Consequently, 19 children (15 male and 4 female; 2 left-handed children) with ASD (mean age = 10.15; *SD* = 1.09; range = 8:6–12:2) and 18 TD children (14 male and 4 female; 2 left-handed children) (mean age = 10.03; *SD* = 1.15; range = 8:5–12:0) were included in analysis. Mean age, AQ scores, and IQ scores are listed in the left column of Table 1. Independent samples *t*-tests showed that the groups were matched for age [*t*_(35)_ = −0.309, *p* = 0.759], and Full scale IQ [*t*_(35)_ = 0.740, *p* = 0.464]. AQ scores showed a significant difference between groups [*t*_(35)_ = −11.49, *p* < 0.001]. The parents of all the participants gave written informed consent to participate in this study, which was conducted in accordance with the institutional ethics provisions.

**Table 1:** **Mean (SD; range) chronological age, IQ scores, and AQ scores from all participants (left column) and from the participants who were included in analysis in the recognition task (right column) for each ASD and TD group**.

	**All participants**			**Participants analyzed in the recognition task**		
	**TD**	**ASD**	***t*-value**; ***p*-value**	**TD**	**ASD**	***t*-value; *p*-value**
Sex	Male = 14; Female = 4	Male = 15; Female = 4	–	Male = 11; Female = 3	Male = 8; Female = 2	–
Handedness	Left-handed = 2	Left-handed = 2	–	Left-handed = 2	Left-handed = 1	–
Age	10.03 (1.15)	10.15 (1.09)	*t*_(35)_ = −0.309	10.09 (1.30)	10.47 (1.10)	*t*_(22)_ = −0.775
	(8:5–12:0)	(8:6–12:2)	*p* = 0.759	(8:5–12:0)	(8:7–12:2)	*p* = 0.447
Full-scale	105.7 (13.37)	102.3 (15.06)	*t*_(35)_ = 0.740	103.3 (9.28)	103.4 (13.85)	*t*_(22)_ = −0.023
IQ	(89–148)	(73–124)	*p* = 0.464	(89–118)	(88–121)	*p* = 0.982
AQ	13.06 (3.33)	29.58 (5.16)	*t*_(35)_ = −11.49	12.5 (3.50)	28.0 (4.92)	*t*_(22)_ = −8.53
	(7–17)	(22–40)	*p* < 0.001	(7–17)	(22–35)	*p* < 0.001

### Apparatus

Visual stimuli were presented on a 15-inch touch-sensitive monitor with a resolution of 1024 by 768 pixels (Mitsubishi, RDT151TU or TSD-AT1515-CN), controlled by custom-written software under Visual Basic 2010 (Microsoft Corporation, Redmond, Washington, USA) running on a personal computer (HP Compaq 6730b/CT or Panasonic CF-SX2).

### Stimuli

#### Warming-up trials

**Figure 1:**
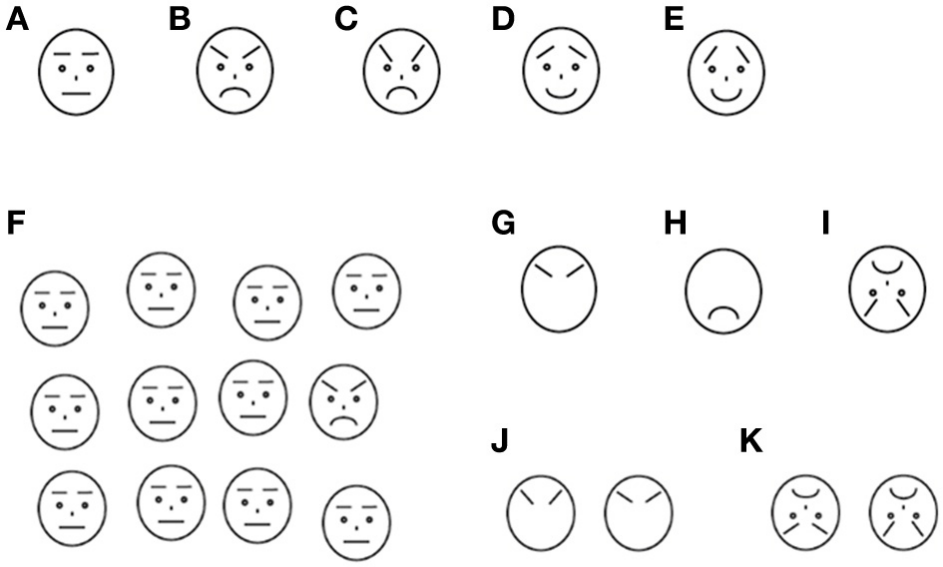
**Stimuli used in this study. (A)** Neutral face **(B)** Angry face with Emotion-degree1 **(C)** Angry face with Emotion-degree2 **(D)** Happy face with Emotion-degree1 **(E)** Happy face with Emotion-degree2 **(F)** Examples of matrix in the search: one angry face with Emotion-degree1 and 11 distractor neutral faces **(G–I)** Examples of the stimuli used in the recognition task; **(G)** Eyebrows of angry face with Emotion-degree1 **(H)** Mouth of angry face with Emotion-degree2 **(I)** Inverted angry face with Emotion-degree2 **(J), (K)** Examples of the choices in the recognition task.

Each trial included the presentation of a self-start key, a fixation picture, and face stimuli. A light-blue-colored rectangle (179 (W) × 136 (H) pixels: 5.3 cm × 4.1 cm on screen (7.6° × 5.9° of visual angle) was used as the self-start key, which was presented at 1.5 cm (2.1° of visual angle) above the bottom of the screen. In the middle of the rectangle, a trial number was presented so that participants could know how many trials they had completed. Fixation pictures were presented at the center of the screen and covering the whole stimulus area of faces. Twenty-four types of pictures of popular cartoon characters were used for the fixation pictures. The face stimuli were schematic pictures portraying angry, happy, and neutral facial expressions. They were created with reference to previous studies (Ashwin et al., 2006; Horstmann, 2007; Isomura et al., submitted). The faces were drawn in black against a white background. All lines in the face drawings were of 2 pixel width. The individual faces were 48 (W) by 54 (H) pixels (1.4 × 1.6 cm on the screen (2.0° × 2.3° of visual angle). Each emotion had two types of faces which were different in the angle of eyebrows and flatness of mouth (Each type was named Emotion-degree1, and Emotion-degree2, respectively) Figures 1(A–E). The face stimuli were presented inside a stimulus area of 268 × 218 pixels (8.0 × 6.5 cm on the screen (11.4 × 9.3° of visual angle). The stimulus area was divided into 4 × 3 grids. We randomized positions of face stimuli for each trial. First we randomly chose a grid for each face stimulus and then altered its position within a grid in a range of ± 8 pixels from the center of the grid in both vertical and horizontal dimensions. This procedure resulted in a moderately irregular arrangement of the stimuli, intended to eliminate possible suprastimulus cues to the target's position (Duncan and Humphreys, 1989; Horstmann, 2007). An example of stimulus displays is shown in Figure 1F.

#### Search-Recognition task

In the main task, named the Search-Recognition task (see procedure for details), recognition task was added to the search task. Stimuli to be recognized were varied in whole faces (identical with the faces used in the search task), local features with outline of face, and inverted faces. Examples of the recognition stimuli were shown in Figures 1G–I.

### Procedure

#### Warming-up trials

First, participants were given 36 trials of a face-in-the-crowd task (i.e., visual search task). This was conducted to calculate individual's mean response time in detection of target faces which would be used in the subsequent Search-Recognition task. Participants were seated approximately 40 cm from the monitor with eye level at the center of the screen, and instructed to touch a discrepant object as quickly and accurately as possible. Each trial started when participants touched the self-start key, after which a fixation picture was presented for 500 ms to keep the children's attention on the screen, and then the face stimuli were displayed. Face stimuli consisted of one emotional face (target) and 11 neutral faces (distractors). The face stimuli were presented until a response was made. When the participants responded correctly, a high tone sounded and a cartoon picture which indicated a correct response was presented, whereas a low tone sounded and a cartoon picture which indicated an incorrect response was presented when they made an incorrect response. Emotion-type (Angry/Happy) and Emotion-degree (1/2) were varied with a pseudorandom sequence. Target position was also controlled by pseudorandom sequences. It took participants approximately 2–3 min to complete all trials.

#### Search-Recognition task

After the warming-up trials, participants were given 6 blocks of the Search-Recognition task. Each block consisted of 36 trials, 12 trials of which were test trials (search-recognition trials) and the rest of the trials were baseline trials (only search trials). In the test trials, the search task was immediately followed by a recognition task where the participants were additionally required to recognize the target face that they had detected in the preceded search task. The recognition task was given only when the participants made a correct choice in the search trial. In the recognition task, whole faces, local-features of faces (i.e., eyebrows or mouth), or inverted faces were presented randomly, and two Emotion-degrees of faces from the same emotion and same Recognition-type [whole faces, local-features of faces (i.e., eyebrows or mouth), or inverted faces] were given as choices. Examples of the display on the recognition trials are shown in Figures 1J,K. They were told that there was a time-limit during the search and thus solve the task as quickly as possible. The time-limit was, however, set only in the baseline trials, and it was not applied to test trials. The time-limit was set individually, with the time calculated by the mean response time in the warming-up trials multiplied by 1.25. If participants could not respond within a given time in the baseline trials, the trial was terminated and visual and auditory feedback which indicated time-out was given. Otherwise, the same visual and auditory feedback as the warming-up trials was given according to their response. The test trials were presented once in 3 trials in average to prevent participants from expecting the presentation of test trials. This less-frequent and random presentation of test trials and time-limit in the baseline trials was employed to avoid participants using the intentional strategy of spending more time to perform better in the recognition task. Some children took a rest between blocks, and in total it took 20–30 min for children to complete all trials.

### Data analysis

Participants' performance on the test trials in the Search-Recognition task was analyzed. Relative accuracy (percentage of correct response to all trials) and median response time on correct trials were calculated individually at each condition separately and used for statistical analysis. Because outliers do not affect the median value as strongly as mean, we did not exclude any values obtained from each participant. All statistical analysis was performed using SPSS 22 (IBM Japan, Ltd).

## Results

**Figure 2:**
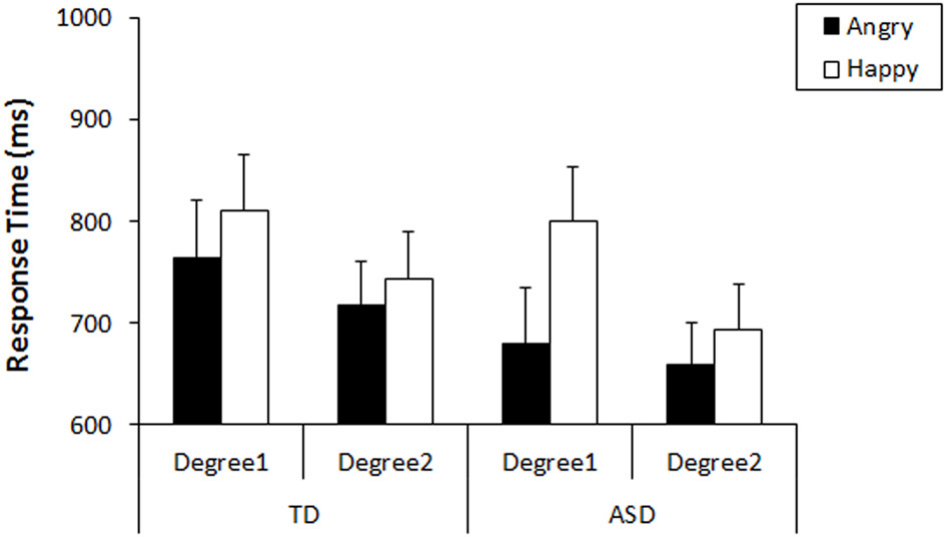
**Mean response times in the detection of angry/happy targets with each Emotion-degree in the search**. Error bars: 95% CI.

First, we analyzed their performance on the search task that preceded the recognition task. As our tasks were designed to produce no or very low numbers of errors, the response times were used for analyses (Results of accuracy were shown in Figure S1). We conducted a general linear model (GLM) repeated measures on the response times with three factors: Emotion-type (Angry vs. Happy), Emotion-degree (Degree1 vs. Degree2), and Group (TD vs. ASD). The results revealed that there was a main effect of Emotion-type [*F*_(1, 35)_ = 26.80, *p* < 0.001, η^2^_*p*_ = 0.434], a main effect of Emotion-degree [*F*_(1, 35)_ = 40.10, *p* < 0.001, η^2^_*p*_ = 0.534], and an interaction between Emotion-type and Emotion-degree [*F*_(1, 35)_ = 9.35, *p* = 0.004, η^2^_*p*_ = 0.211]. Neither main effect of Group [*F*_(1, 35)_ = 2.78, *p* = 0.105, η^2^_*p*_ = 0.074] nor interactions involving Group [Group × Emotion-type: *F*_(1, 35)_ = 3.49, *p* = 0.070, η^2^_*p*_ = 0.091; Group × Emotion-degree: *F*_(1, 35)_ = 0.114, *p* = 0.737, η^2^_*p*_ = 0.003; Group × Emotion-type × Emotion-degree: *F*_(1, 35)_ = 3.63, *p* = 0.065, η^2^_*p*_ = 0.094] reached statistical significance, but trends of group difference was found in interaction with Emotion-type, as well as with the other two factors. Subsequent analysis (Bonferroni correction) for the interaction between Emotion-type and Emotion-degree revealed that angry faces were detected more quickly than happy faces both in the faces of Emotion-degree1 (i.e., less exaggerated), *F*_(1, 35)_ = 24.82, *p* < 0.001, η^2^_*p*_ = 0.415, and in faces of Emotion-degree2 (i.e., more exaggerated), *F*_(1, 35)_ = 8.85, *p* = 0.005, η^2^_*p*_ = 0.202. This indicated that ASE existed in children of both groups, but it was less significant when the faces included more exaggerated features (Figure 2). These results would be explained by exaggerated emotional faces being physically and emotionally more salient among neutral faces compared to the less exaggerated ones, and that it resulted in showing some floor effect on response times for the detection of exaggerated angry faces and exaggerated happy faces. However, the robust phenomenon of faster detection of angry faces than happy faces (i.e., ASE) was observed both in TD and ASD.

**Figure 3:**
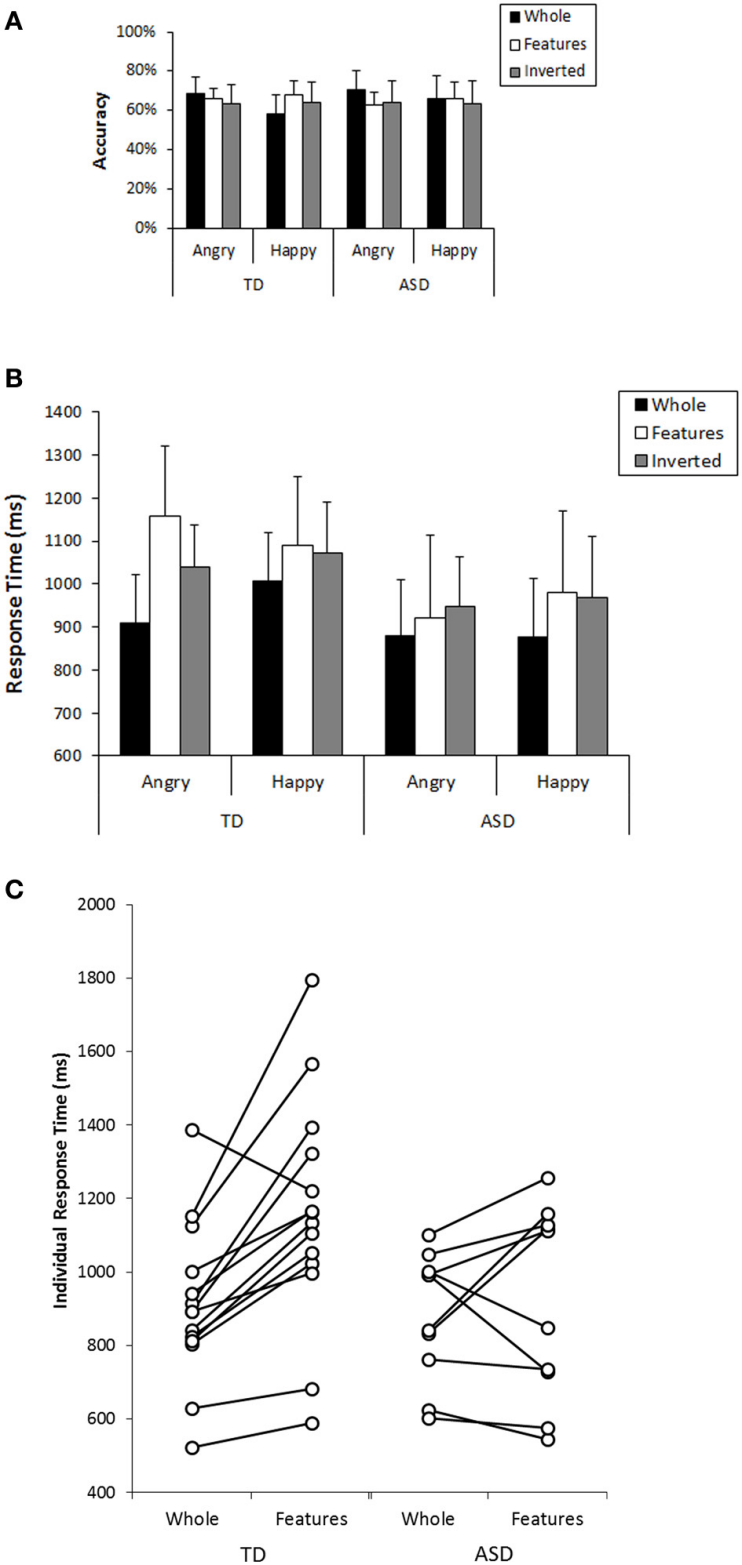
**(A)** Mean accuracy in the recognition task for each condition in TD and ASD group. Error bars: 95% CI. **(B)** Mean response times in the recognition task for each condition in TD and ASD group. Error bars: 95% CI. **(C)** Comparison in the response times between recognition of *angry whole faces* and recognition of *local features in angry faces* in individual subjects in TD and ASD.

Next, we focused on participants' performance in the recognition task that followed the search task to examine their cognitive pattern employed during the search. In analyzing this, participants who could not perform better than expected by chance (a binomial test with significance level of 0.1) in both whole recognition and in features recognition, which were our main focus, were excluded from further analyses because we could not confirm that they understood the task requirement properly. Consequently, 4 individuals from the TD group and 9 individuals from the ASD group were excluded from the subsequent analysis (Mean accuracy and mean response time at each condition from all participants are shown in Figure S2). Information from the participants who were included in this analysis was listed in the right column of Table 1. Participants' performance (mean values, SDs, and 95%CIs for accuracy and response times in each group) was described in Table 2. We conducted a GLM analysis with repeated measures on the accuracy data with Recognition-type (Whole, Features vs. Inverted), Emotion (Angry vs. Happy), as the within-subjects factor, and Group (ASD vs. TD) as the between-subjects factor. The results revealed neither main effects nor significant interactions [Recognition-type: *F*_(2, 44)_ = 0.321, *p* = 0.727, η^2^_*p*_ = 0.014; Emotion: *F*_(1, 22)_ = 0.398, *p* = 0.535, η^2^_*p*_ = 0.018; Group: *F*_(1, 22)_ = 0.037, *p* = 0.849, η^2^_*p*_ = 0.002; Recognition-type × Emotion: *F*_(2, 44)_ = 2.35, *p* = 0.108, η^2^_*p*_ = 0.096; Recognition-type × Group: *F*_(2, 44)_ = 0.929, *p* = 0.403, η^2^_*p*_ = 0.040; Emotion × Group: *F*_(1, 22)_ = 0.141, *p* = 0.711, η^2^_*p*_ = 0.006; Recognition-type × Emotion × Group: *F*_(2, 44)_ = 0.281, *p* = 0.756, η^2^_*p*_ = 0.013] (Figure 3A). We further conducted a GLM analysis with repeated measures on response times with the same three factors above: Recognition-type, Emotion, and Group. The results revealed a main effect of Recognition-type [*F*_(2, 44)_ = 9.701, *p* < 0.001, η^2^_*p*_ = 0.306], and a three-way interaction among all factors [*F*_(2, 44)_ = 3.94, *p* = 0.027, η^2^_*p*_ = 0.152]. Other factors did not show any statistically significant effect [Emotion: *F*_(2, 44)_ = 1.59, *p* = 0.215, η^2^_*p*_ = 0.068; Group: *F*_(1, 22)_ = 1.83, *p* = 0.190, η^2^_*p*_ = 0.077; Recognition-type × Group: *F*_(2, 44)_ = 1.59, *p* = 0.215, η^2^_*p*_ = 0.068; Emotion × Group: *F*_(1, 22)_ = 0.032, *p* = 0.860, η^2^_*p*_ = 0.001; Recognition-type × Emotion: *F*_(2, 44)_ = 0.791, *p* = 0.460, η^2^_*p*_ = 0.035]. Subsequent analysis for three-way interaction showed that there was a significant simple interaction between Group and Recognition-type when the Emotion was Angry [*F*_(2, 44)_ = 4.61, *p* = 0.015, η^2^_*p*_ = 0.173], but no simple interaction was found when the Emotion was Happy [*F*_(2, 44)_ = 0.076, *p* = 0.927, η^2^_*p*_ = 0.003]. This indicated that the Group difference was observed only for recognition of angry faces, but not for recognition of happy faces. Further analysis in the recognition of angry faces revealed a simple simple main effect of the Recognition-type in TD [*F*_(2, 26)_ = 13.03, *p* < 0.001, η^2^_*p*_ = 0.501], but not in ASD [*F*_(2, 18)_ = 1.04, *p* = 0.373, η^2^_*p*_ = 0.104]. Subsequent multiple comparisons (Bonferroni correction) in the TD group revealed that recognition of whole faces showed shorter response time than recognition of inverted faces or recognition of local features (*p* = 0.001, *p* = 0.002, respectively), but there was no significant difference on the response times between the recognition of inverted faces and the recognition of local features (*p* = 0.183) (Figure 3B). These results indicated that TD children showed better performance in the recognition of angry whole faces than in the recognition of local features in angry faces or angry inverted faces, whereas ASD children showed similar performance among them. Even though the sample size may not be sufficient to clearly reveal the group differences, consistent tendency was observed in the most of individuals within each group. The comparison between response times on recognition of angry whole faces and recognition of local features in angry faces are shown in Figure 3C.

**Table 2:** **Accuracy and response times (means, standard deviations, and 95% CIs) for each group at each condition in recognition**.

		**Angry**									**Happy**								
		**Whole**			**Features**			**Inverted**			**Whole**			**Features**			**Inverted**		
		**Mean**	**SD**	**95%CI**	**Mean**	**SD**	**95%CI**	**Mean**	**SD**	**95%CI**	**Mean**	**SD**	**95%CI**	**Mean**	**SD**	**95%CI**	**Mean**	**SD**	**95%CI**
TD	Accuracy	68.9%	3.9%	60.9%	65.8%	2.7%	60.2%	63.7%	4.4%	54.5%	58.4%	4.5%	49.0%	68.0%	3.4%	61.0%	64.3%	4.8%	54.4%
				~77%			~71.5%			~72.9%			~67.8%			~74.9%			~74.2%
	Response Time (ms)	910.1	53.6	799.0	1158.3	78.5	995.4	1039.4	47.5	941.0	1006.2	54.7	892.8	1090.6	77.3	930.3	1071.1	57.4	952.1
				~1021.3			~1321.1			~1137.9			~1119.7			~1250.8			~1190.0
ASD	Accuracy	70.8%	4.6%	61.3%	62.6%	3.2%	55.9%	64.3%	5.3%	53.4%	66.2%	5.4%	55.0%	66.0%	4.0%	57.8%	63.6%	5.6%	51.8%
				~80.2%			~69.3%			~75.2%			~77.3%			~74.2%			~75.3%
	Response Time (ms)	879.6	63.4	748.0	920.4	92.9	727.7	947.3	56.2	830.8	878.3	64.7	744.0	981.3	91.4	791.7	969.0	67.9	828.2
				~1011.1			~1113.0			~1063.7			~1012.5			~1170.9			~1109.7

In addition, the results of ANOVA showed another simple interaction between Recognition-type and Emotion [*F*_(2, 26)_ = 4.05, *p* = 0.030, η^2^_*p*_ = 0.237] in TD. In ASD, no simple interaction was found [*F*_(2, 18)_ = 0.763, *p* = 0.481, η^2^_*p*_ = 0.078]. Further analysis (Bonferroni correction) in the TD group showed that the recognition of angry whole faces showed faster response time than the recognition of happy whole faces [*F*_(1, 13)_ = 5.01, *p* = 0.043]. On the other hand, the recognition of local features in angry faces showed marginally significant longer response time than that in happy faces [*F*_(1, 13)_ = 3.21, *p* = 0.097] (Figure 3B). These results indicated that TD children showed better performance in recognizing angry faces than in recognizing happy faces when they were presented as whole faces, but the opposite tendency was observed when they were presented as local features. In ASD, such tendency was not observed.

## Discussion

The current study revealed that ASE exists in individuals with ASD as well as the TD individuals, consistent with previous studies (Ashwin et al., 2006; Krysko and Rutherford, 2009; Rosset et al., 2011; Isomura et al., submitted). More importantly, we obtained evidence that different mechanisms may underlie ASE between ASD children and TD children. The results of the recognition task revealed that TD children and ASD children processed particularly angry faces in different manners. TD children took more time to recognize local features in angry faces and angry inverted faces than to recognize angry whole faces. Furthermore, shorter time was required for the recognition of angry whole faces than in the recognition of happy whole faces, on the other hand, longer time was required in the recognition of local features in angry faces than in happy faces. These results suggest that detection of angry faces required more configural face processing and disrupted the processing of local features in TD children, as we had hypothesized. This is consistent with previous studies that revealed negative facial expressions capture attention and disrupt the processing of local features (Eastwood et al., 2003, 2008). In ASD, however, they showed similar response times among the recognition of whole faces, local features and inverted faces in angry faces. This suggests that detection of angry faces in ASD was processed in a feature-based manner rather than configural processing. Although the sample sizes were small, our results provide the preliminary evidence that they may, in contrast to TD children, extract facial information from local features, but still showed rapid processing of angry faces over happy faces similarly to TD children. This suggested the possibility that local features in angry faces by themselves may work as emotion-evoking stimuli that elicit rapid processing for children with ASD, in contrast to TD individuals where rapid processing of angry faces does not emerge from single feature detection (Fox et al., 2000; Tipples et al., 2002; Weymar et al., 2011).

Previous studies on facial emotion recognition have reported that individuals with ASD use local, feature-based processing, in contrast to the global, configural-based strategy used by TD individuals (Tantam et al., 1989; Behrmann et al., 2006; Harms et al., 2010). Furthermore, some evidence suggests that individuals with ASD may interpret emotional faces by memorizing the specific features associated with each emotion (i.e., rule-based strategy; Rutherford and McIntosh, 2007; Harms et al., 2010). The results in the current study revealed that the feature-based processing in ASD caused faster detection of angry faces over happy faces, even if they compensatorily learn how to interpret emotional faces. Taken together with the previous finding that revealed the age-related acquisition of ASE in individuals with ASD (Isomura et al., submitted), we propose the following hypothesis on mechanisms behind ASE in individuals with ASD. Individuals with ASD may not show innate mechanisms to orient toward angry faces rapidly, because they failed to treat angry faces as threatening stimuli. However, as they compensatorily learn the way to interpret facial emotions and become able to connect angry facial expressions to threat, they may start to show proper emotional responses that were observed in ASE. Further studies are required to examine this possibility.

Finally, several limitations of our study should be acknowledged. First of all, because of the small sample sizes, it would be still early to draw definitive conclusion, especially on the processing style in children with ASD. However, the findings in the current study are in line with previous studies that have suggested feature-based face processing in ASD (Behrmann et al., 2006), and that have reported the less robust effect of the anger superiority, suggesting different processing mechanisms underlie in individuals with ASD (Ashwin et al., 2006; Krysko and Rutherford, 2009). We believe that our exploratory results here have paved a road for future investigations with larger sample sizes. Physiological measurements in addition to behavioral measures would provide more in-depth insight. Second, in the current study, we used schematic faces as stimuli to control low-level perceptual variations. At the same time, however, schematic faces reduced ecological validity. Especially for people with ASD, ecological validity is important because they may develop and apply rules to schematic face stimuli to compensate for their difficulties with emotional detection (Rutherford and McIntosh, 2007). Also, the use of schematic stimuli may have facilitated children with ASD to focus on local features. To confirm the results of the current study, and to examine whether there are differences from the results obtained here using schematic stimuli, we should examine the effect in children with and without ASD using photographic faces. Third, in the current study, we could not examine sex differences because of the small sample size of female participants. As a previous study reported attentional bias toward facial emotions to be different between male and female (Tran et al., 2013), we need to examine the effect of sex in future studies. Moreover, we have only included participants who have normal-range intelligence. To better understand the ASD population as a whole, it is necessary to examine ASE in lower-functioning ASD, which may provide important cues for identifying subtypes of ASD.

## Conclusions

This study demonstrated that the detection of angry faces required more configural face processing and disrupted the processing of local features than the detection of happy faces in TD children, according to the response times in the recognition of faces. In ASD children, on the other hand, the detection of angry faces required feature-based processing rather than configural processing. Despite the small sample sizes, these findings provide the preliminary evidence that different mechanisms underlie both TD and ASD children though they similarly showed faster detection of angry faces over happy faces. In contrast to TD children, children with ASD may extract emotional information from local features in angry faces (i.e., v-shaped eyebrows and downward mouth) and showed the proper emotional response of detecting angry faces over happy faces.

### Conflict of interest statement

The authors declare that the research was conducted in the absence of any commercial or financial relationships that could be construed as a potential conflict of interest.
