# Supplementary material for: Preliminary evidence that different mechanisms underlie the anger superiority effect in children with and without Autism Spectrum Disorders
Source: Front Psychol. 2014 May 27;5:461. doi: 10.3389/fpsyg.2014.00461 (PMC4034349; doi:10.3389/fpsyg.2014.00461)
Supplement: Supplementary file 1 [file DataSheet1.DOCX]

**Frontiers in Psychology: Personality Science and Individual Differences**

Supplementary Material

20 April 2014

**Supplementary Material**

Preliminary evidence that different Mechanisms Underlie the Anger Superiority Effect in Children with and without Autism Spectrum Disorders

Tomoko Isomura^1^*, Shino Ogawa^1^, Satoko Yamada^1^, Masahiro Shibasaki^1^ and Nobuo Masataka^1^

^1^Primate Research Institute, Kyoto University, Inuyama, Japan

Correspondence:

Tomoko Isomura

Primate Research Institute, Kyoto University

41-2 Kanrin, Inuyama, Aichi, Japan

Tel: +81-568-63-0555; Fax: +81-568-62-9552
Email: [isomura.tomoko.35m@st.kyoto-u.ac.jp](mailto:isomura.tomoko.35m@st.kyoto-u.ac.jp)

**Supplementary Figures**

**Figure S1**

Mean accuracy in the detection of angry/ happy targets with each Emotion-degree in the search. Error bars: 95% CI.

**Figure S2**

Mean accuracy (A) and mean response time (B) at each condition in the recognition task from all participants. Error bars: 95% CI.
